# Differential Involvement of Lateral Semicircular Canal and Otolith Organs in Common Vestibular Disorders

**DOI:** 10.3389/fneur.2022.819385

**Published:** 2022-01-31

**Authors:** Yehree Kim, Byung Chul Kang, Myung Hoon Yoo, Hong Ju Park

**Affiliations:** ^1^Department of Otorhinolaryngology-Head and Neck Surgery, Asan Medical Center, University of Ulsan College of Medicine, Seoul, South Korea; ^2^Department of Otorhinolaryngology-Head and Neck Surgery, Ulsan University Hospital, University of Ulsan College of Medicine, Ulsan, South Korea; ^3^Department of Otorhinolaryngology-Head and Neck Surgery, School of Medicine, Kyungpook National University, Daegu, South Korea

**Keywords:** dizziness, vertigo, head impulse test, otolith, vestibular-evoked myogenic potential (VEMP), benign paroxysmal positional vertigo (BPPV), Meniere disease, vestibular neuritis (VN)

## Abstract

Semicircular canal and otolith functions came to be evaluated recently, but comprehensive and comparative analysis of canal and otolith dysfunction in common vestibular disorders is lacking. We aimed to analyze the abnormal rates of canal and otolith function in common vestibular disorders. We enrolled 302 patients who were managed for 2 months in a dizziness clinic. Results of caloric, video head impulse test (vHIT), and cervical and ocular vestibular evoked myogenic potential (cVEMP and oVEMP) tests were analyzed and compared among various diagnoses. Vestibular disorders diagnosed included recurrent vestibulopathy (RV, 27%), vestibular migraine (VM, 21%), benign paroxysmal positional vertigo (BPPV, 17%), Meniere's disease (MD, 11%), vestibular neuritis (VN, 10%), orthostatic dizziness (7%), and central lesions (3%). Lateral canal dysfunction was found most in VN (100%) and less commonly in definite MD (75%), RV (46%) and definite VM (29%). Abnormal caloric results were more common than abnormal vHIT in all disorders. Otolith dysfunction was found more frequently than lateral canal dysfunction in most vestibular disorders except VN. An abnormal cVEMP was more frequent in definite MD than the other disorders. Isolated otolith dysfunction without lateral canal dysfunction was the most found in BPPV, followed by definite VM, RV, and definite MD in decreasing order. Various patterns of involvement in canal and otoliths were revealed in vestibular disorders, suggesting different pathogenesis.

## Introduction

Based on a report from a national survey in the United States, vestibular dysfunction is a common condition with a 4-year prevalence of 35.4% in adults aged 40 years and older (1). Dizziness is one of the most common complaints in an outpatient neurotologic clinic and the epidemiology of vestibular disorders is of interest when evaluating these cases (2–4). It can be easier for a physician to think of the most common diseases as a cause of dizziness but a definitive diagnosis of dizziness is still made through a detailed history taking and thorough examination. In general, the vestibular disorders benign paroxysmal positional vertigo (BPPV), Meniere's disease (MD), and vestibular neuritis (VN) have been considered the most common causes of dizziness. Recently however, there is increasing recognition of the role of a syndrome known as vestibular migraine (VM) as it affects more than 1% of the general population but about 10% of patients in dizziness clinics and at least 9% of patients in migraine clinics (5).

To evaluate the vestibular dysfunction, caloric stimulation is the most commonly used classical method to quantify and identify the side of peripheral vestibular dysfunction. Recently, there has been significant recent progress in the evaluation of vestibular function. The function of the lateral semicircular canal can now be assessed at different frequency ranges. The video head impulse test (vHIT) assesses the function of the semicircular canals in response to high-frequency head movements and is therefore a more suitable method for assessing the real-life vestibular function of the canals compared to the caloric test (6–8). The cervical and ocular vestibular evoked myogenic potential (cVEMP and oVEMP) tests assess the function of otolith organs (saccule and utricle), respectively (9, 10). Thus, the caloric result in a normal range of a dizzy patient does not mean that the vestibular function of the patient is normal, because the caloric test evaluates only the lateral canal function and does not provide an overall vestibular function. There have been some studies that have analyzed the epidemiology of vestibular disorders, but few reports to date have comprehensively compared the results of vestibular function tests in patients in a dizziness clinic (2–4). We speculated that a comparison of the results of each test in patients with different vestibular disorders might improve our understanding of the involvement patterns of vestibular organs in those different conditions.

We investigated the epidemiological features of patients with dizziness in an outpatient clinic and compared the results of vestibular function tests (VFTs) to investigate how differently the vestibular organs are involved in various vestibular disorders.

## Materials and Methods

A retrospective chart review identified a cohort of 302 patients with a chief complaint of dizziness and an age above 15, who were managed in a dizziness clinic from October to November 2014. The diagnoses were finalized by considering previous or follow-up clinical results for these patients. We performed vestibular function tests (VFTs) during an interictal period, which was usually within 10 days after the first visit to the clinic. Because not all of the patients underwent VFTs, we analyzed and compared the VFT results from the cases who were diagnosed with the most frequent five vestibular disorders. This study protocol was approved by the institutional review board of the institute.

### Diagnosis of Vestibular Disorders

The study patients were diagnosed following the criteria for vestibular disorders listed in Table 1. A benign paroxysmal positional vertigo (BPPV) diagnosis was based on clinical history and physical findings (11, 19). Recurrent vestibulopathy (RV) is a disease that displays recurrent symptoms of episodic vertigo that last for several minutes to several hours without auditory or neurologic signs (13), whereas MD symptoms include episodic vertigo, fluctuant hearing loss, aural pressure and tinnitus (15). Vestibular neuritis (VN) was diagnosed if the patient had a previous acute onset of severe prolonged vertigo lasting >24 h without hearing loss or other neurologic symptoms and also caloric irrigation showing a decreased responsiveness in the affected ear (canal paresis > 20%). A diagnosis of VM was based on the recently reported criteria for VM (14), which involve at least five episodes of vestibular symptoms of moderate or severe intensity lasting between 5 min and 72 h, a current or previous history of migraine with or without aura in accordance with the International Classification of Headache Disorders criteria, and one or more migraine features occurring with at least 50% of the vestibular episodes. Orthostatic dizziness (OD) is defined as dizziness provoked by orthostatic positional change, such as standing upright from a supine or sitting position (12). Any patients with a persistent or long-lasting history of dizziness or physical findings suggesting central causes were evaluated by MRI with intravenously injected gadolinium to rule out a brain tumor or diffusion MRI to rule out a stroke. The references listed in Table 1 provide detailed criteria for all of these diagnoses.

**Table 1:** Diagnostic clues to the disorders causing dizziness from the clinical features and common findings in the affected patients.

**Diagnoses**	**Clues from the patient history**	**Common findings from the physical examinations**
Positional vertigo/dizziness: occur mostly from head movements		
BPPV (11)	Transient vertigo <1 min when rolling, rising up, or lying down when in bed	Canal-specific nystagmus in Dix-Hallpike, roll, or bow-and-lean tests according to the types of BPPV
Orthostatic dizziness (12)	Recent change or new medication, old age, occurs mostly when rising up or after exercise, and from dehydration	Occurrence of dizziness upon an abrupt sitting up but not while lying down or rolling; BP check in supine & upright positions
Spontaneous vertigo/dizziness: can occur regardless of head movements		
Recurrent vestibulopathy (13)	Recurrence, no other neurologic symptoms	n.s.
Vestibular migraine (14)	Co-occurrence of headache before/during/after vertigo or dizziness	n.s.
Vestibular neuritis	Single episode of vertigo for more than 24 h & subsequent imbalance, no other neurologic symptoms	Positive bedside head impulse test, impaired but not severe imbalance in the Romberg test
Meniere's disease (15)	Co-occurrence of hearing loss or tinnitus or ear-fullness with vertigo	Positive bedside head impulse test (rarely), Weber test: lateralization to the contralateral ear when hearing loss is present.
Acute central vertigo (16)	Associated with other neurologic symptoms	Abnormal neurologic examination, positive HINTS, severe postural instability in the Romberg test
Vestibular schwannoma	Unilateral progressive hearing loss or tinnitus, imbalance	Positive bedside head impulse test; Weber test: lateralization to the contralateral ear
Superior canal dehiscence syndrome (17)	Unilateral autophony, pulsatile tinnitus, hyperacusis, ear-fullness, sound/pressure-induced dizziness	Weber test: lateralization to the ipsilateral ear, dizziness or nystagmus from noise or pressure to the ipsilateral ear, or a Valsalva maneuver
Vestibular paroxysmia (18)	A high frequency of vertigo attacks (up to 30/day) for seconds or minutes, typewriter tinnitus	Dizziness or nystagmus caused by hyperventilation

*BPPV, benign paroxysmal positional vertigo; BP, blood pressure; HINTS, Head-Impulse-Nystagmus-Test-of-Skew; n.s, nonspecific*.

### Vestibular Function Tests

We analyzed the VFT results in patients with specific vestibular disorders who underwent vestibular function tests including vHIT, cervical and ocular VEMP, and caloric tests. The breakdown of the patients who underwent these tests was as follows: 30 vestibular neuritis, 22 definite Meniere's disease, 54 recurrent vestibulopathy, and 35 definite vestibular migraine cases. In 39 patients with BPPV, a caloric test was not performed but vHIT and VEMP tests were conducted and the results analyzed.

The bithermal caloric test was used in our study patients and eye movements were recorded using a video-based system (ICS water caloric stimulator NCI-480; Otometrics, Denmark). Each ear was irrigated with a constant flow of water at temperatures at 30°C and 44°C for 30 s. The maximum slow-phase eye velocities of the nystagmus were calculated after each irrigation session. The Jongkees formula was used to determine canal paresis, and was considered pathologic at 20% or more.

The function of the lateral canal was also assessed by rotating the head in the horizontal plane in unpredictable directions using an ICS Impulse 3-D vHIT system (GN Otometrics; Taastrup, Denmark). We evaluated vHIT gains and gain asymmetry in the lateral semicircular canal plane. Each vHIT gain was calculated from the ratio of the area under the curve (AUC) for the eye movement divided by the AUC for the head movement. Gain asymmetry (GA) was calculated from the gains obtained in response to rightward and leftward head impulses in accordance with the following equation: GA = [(Gc – Gi)/(Gc + Gi)] × 100%; where Gc is the vHIT gain exciting the contralateral lateral canal and Gi is the vHIT gain exciting the ipsilateral lateral canal (the side with low vHIT gain). The corrective saccades (CSs) were classified as covert if they occurred before the end of the head movement, or as overt if the onset was after the end of the head movement. The occurrence of CS was defined as three or more CS incidents of similar amplitudes, thus differentiating an actual CS from an artifact. A vHIT gain of ≤0.8, or GA ≥8%, or a CS peak velocity of ≥100°/s was considered pathologic, as described previously (20).

Cervical VEMPs were recorded in the sitting position, with the head rotated away from the stimulated side during recording. The surface electrodes were placed as follows: the active electrode was placed over the middle third of the sternocleidomastoid muscle, the reference electrode was placed on the upper sternum, and the ground electrode was placed on the forehead. VEMPs were elicited using 500-Hz Blackman tone pips with a 2-ms rise/fall time and 1-ms plateau presented at a rate of 9 per second through insert earphones. The stimulus intensities were 90 dB nHL, and the electromyography signal was amplified and bandpass filtered (30-1,500 Hz) using the GSI Audera system (Grason-Stadler, Eden Prairie, MN). For oVEMP test, the active electrodes were placed on the face just inferior to the contralateral eye to the sound stimulation, around 1 cm vertically oriented below the center of the lower eyelid, the reference electrode about 1 cm below the active one on the cheek, and the ground electrode on the forehead. The subjects were seated in a comfortable chair. During recording, the subject was instructed to look superiorly at a small fixed target 1 m from the eyes, with a visual angle of approximately 30°. We analyzed the peak-to-peak amplitude of the N1 and P1 at the maximal intensity of stimulation. The VEMP results were considered pathological if there was an interaural amplitude difference ratio greater than 40%, an interaural difference in threshold >15 dB, or an absence of VEMP (21).

### Statistical Analysis

Data were expressed as the mean ± standard deviation. Caloric, vHIT, and VEMP test results were compared against each other in the same subgroup of vestibular disorders (inter-test comparison in the same vestibular disorder, e.g., caloric vs. vHIT, cVEMP vs. oVEMP) and between the subgroups with different vestibular disorders (inter-disorder comparison). Categorical variables were compared using the Fisher's exact test. All *p* < 0.05 were considered significant. All statistical analyses were conducted using SPSS version 18.0 (SPSS software, SPSS Inc., Chicago, IL).

## Results

### Diagnoses of Vestibular Disorders Causing Dizziness

Table 2 presents the frequency of each diagnosis in patients with vertigo who visited our dizziness clinic over a 2-month period (*n* = 302). The most common final diagnoses assigned to this population of patients were recurrent vestibulopathy (26.5%), followed by vestibular migraine (20.5%), BPPV (17.2%), Meniere's disease (10.6%), vestibular neuritis (10.3%), orthostatic dizziness (7.0%), central lesions (3.3%), and trauma-related dizziness (3.0%).

**Table 2:** Incidence of diagnoses causing dizziness among the 302 dizzy patients in a neurotologic outpatient clinic.

**Diagnoses**	**Female (%)**	**Male (%)**	**Age (yr)**	**No**.	**%**
Recurrent vestibulopathy (RV)	46 (58%)	34 (42%)	59 ± 14	80	26.5
Vestibular migraine (VM)	53 (85%)	9 (15%)	50 ± 15	62	20.5
Benign paroxysmal positional vertigo (BPPV)	34 (65%)	18 (35%)	57 ± 12	52	17.2
Meniere's disease (MD)	20 (63%)	12 (37%)	53 ± 12	32	10.6
Vestibular neuritis (VN)	12 (39%)	19 (61%)	59 ± 13	31	10.3
Orthostatic dizziness	16 (76%)	5 (24%)	59 ± 16	21	7.0
Central lesions (including three vertebro-basilar insufficiency, two infarcts, two cerebello-pontine angle tumors, one Parkinson's disease, one vestibular paroxysmia, one inflammatory pseudotumor)	5 (50%)	5 (50%)	63 ± 16	10	3.3
Trauma-related dizziness (including one perilymph fistula)	7 (78%)	2 (22%)	47 ± 9	9	3.0
Ramsay-Hunt Syndrome	1 (33%)	2 (67%)	61 ± 10	3	1.0
Bilateral vestibular hypofunction	0	2 (100%)	71 ± 4	2	0.7
Total	194 (64%)	108 (36%)	56 ± 14	302	100.0

*Plus–minus values denote a mean ± SD*.

The central lesions identified as the cause of dizziness in some patients included a vertebra-basilar insufficiency in three cases, and an acute infarct in two, cerebello-pontine angle tumor in two, Parkinson syndrome in one, inflammatory pseudotumor in one, and vestibular paroxysmia in one patient. Vertebra-basilar insufficiency was diagnosed *via* magnetic resonance angiography in patients with orthostatic dizziness which was persistent or associated with other neurologic symptoms. Acute infarct was diagnosed in patients with acute vertigo syndrome and positive results in HINTS test or severe postural imbalance in Romberg test.

We examined the frequency of peripheral sources of dizziness vs. non-peripheral sources although they can be mixed in nature. We found that 86.7% of the diagnoses in our current study cohort were peripheral and 3.3% were central when we considered vestibulopathy as a peripheral etiology and orthostatic dizziness and trauma-related dizziness as mixed lesions.

### Comparison of Abnormal VFTs in Vestibular Disorders

Overall, the rates of abnormal findings in canal or otolithic function tests were highest in the VN patients (100%) and decreased in frequency in the following sequence, definite MD (94%), RV (65%), definite VM (62%) and BPPV (56%).

**Figure 1:**
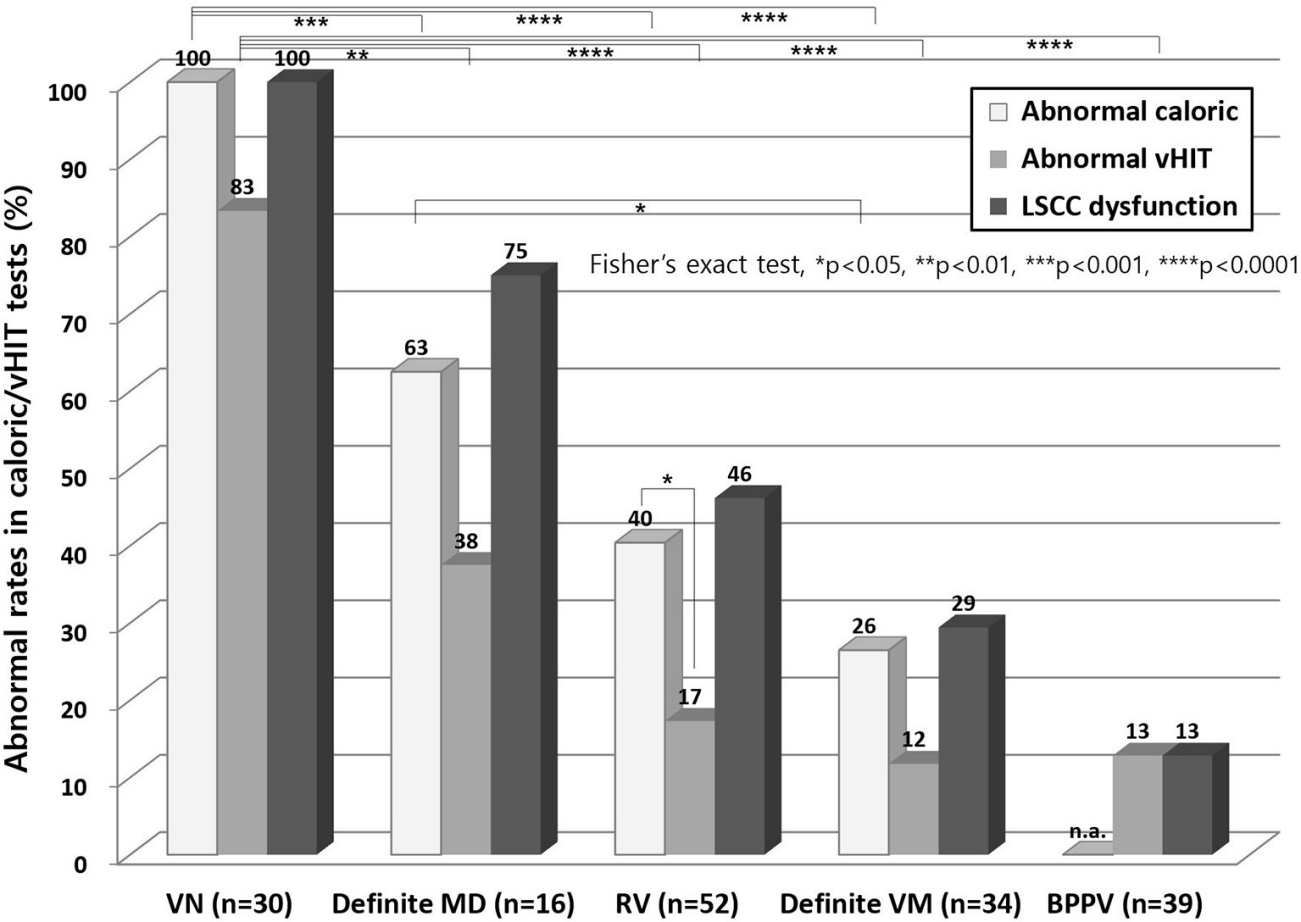
Abnormal results from caloric test and vHITs. Abnormal caloric results were more common than abnormal vHIT findings in all vestibular disorders, although this was a significant finding only in RV. The abnormal caloric and vHIT results were highest in VN and decreased in the following sequence, definite MD, RV and definite VM. vHIT, video head impulse test; LSCC, lateral semicircular canal; n.a, not applicable; VN, vestibular neuritis; MD, Meniere's disease; RV, recurrent vestibulopathy; VM, vestibular migraine; BPPV, benign paroxysmal positional vertigo.

The incidence of abnormal caloric and vHIT results was highest in the VN patients (100% in caloric and 83% in vHIT) and decreased in frequency in the following sequence, definite MD (63 and 38%), RV (40 and 17%) and definite VM (26 and 12%, Figure 1). Abnormal caloric results were more common than an abnormal vHIT in all vestibular disorders, although this was significant only in RV (40 vs. 17%, Figure 1).

**Figure 2:**
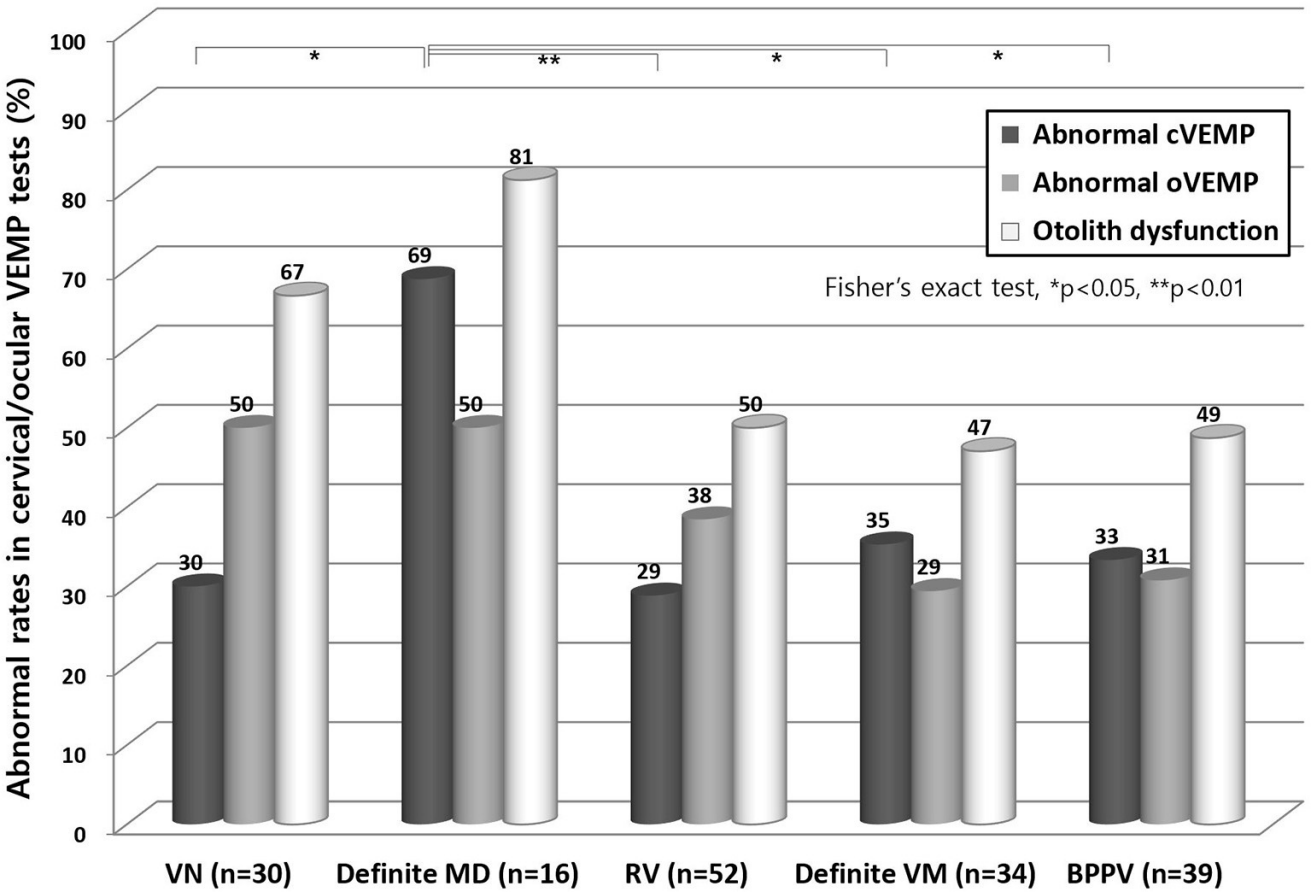
Abnormal results from cervical VEMP and ocular VEMP test. In the cVEMP test, patients with a definite MD showed a higher abnormal rate than with any other disorder (*p* < 0.05). In the oVEMP test, VN and definite MD cases showed the highest degree of abnormality, but this was not significantly different from other disorders. VN, vestibular neuritis; MD, Meniere's disease; RV, recurrent vestibulopathy; VM, vestibular migraine; BPPV, benign paroxysmal positional vertigo; cVEMP, cervical vestibular evoked myogenic potential; oVEMP, ocular VEMP.

In the cVEMP test (Figure 2), definite MD showed the highest abnormal rate (69%) and this was significantly higher than other disorders including VN, RV, definite VM, and BPPV (*p* < 0.05). In the oVEMP test, VN and definite MD showed higher abnormal rates than the other disorders, but not significantly. An abnormal oVEMP was more common than abnormal cVEMP in patients with VN and RV, but the opposite was observed in patients with definite MD, definite VM, and BPPV, although again this was not significant.

**Figure 3:**
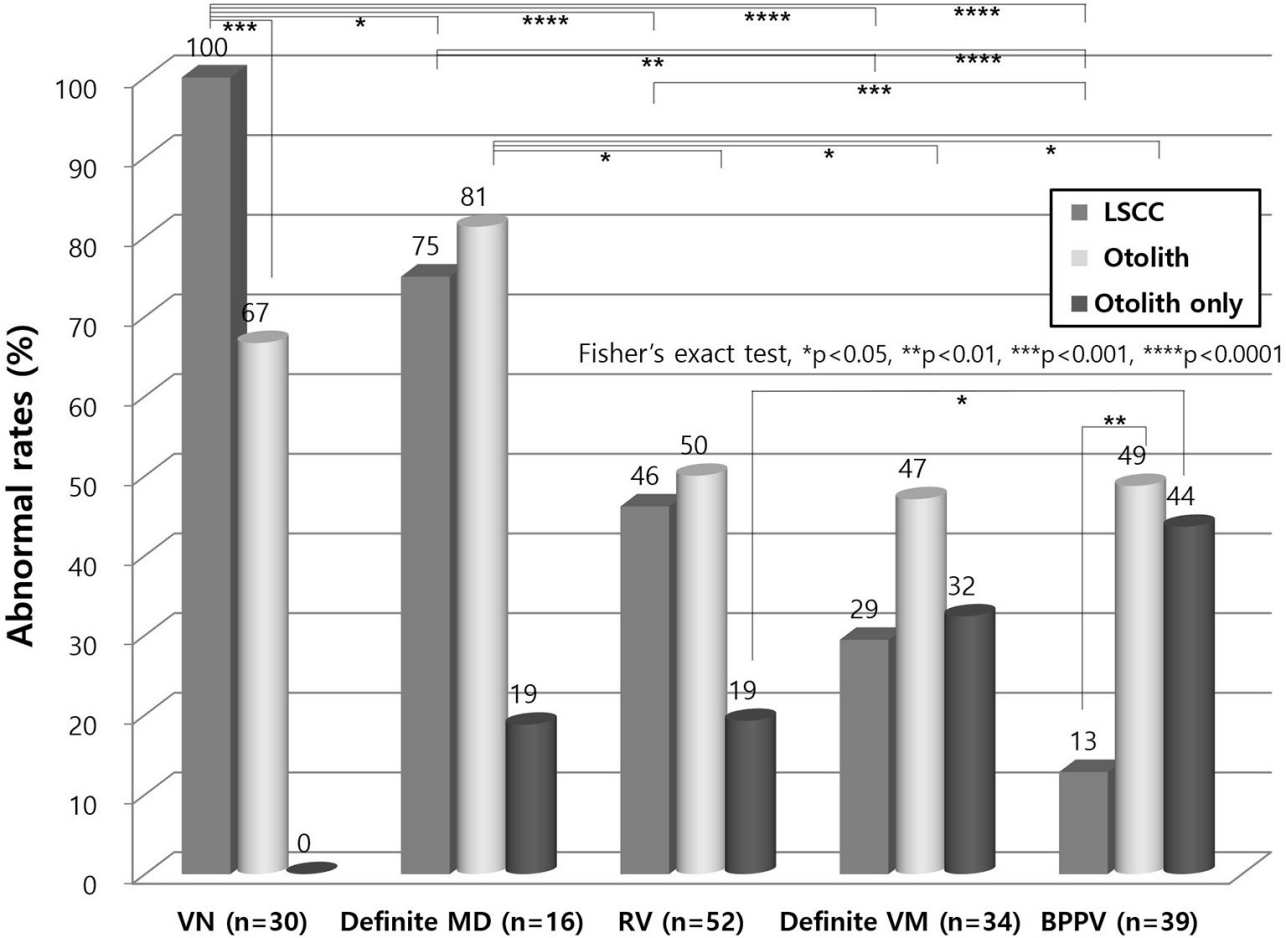
Lateral canal and otolith dysfunction in vestibular disorders. Lateral canal dysfunction was more common than otolith dysfunction in VN (*p* < 0.001). Other vestibular disorders showed higher otolith dysfunction than lateral canal dysfunction which was a significant difference in patients with BPPV (*p* < 0.01). Isolated otolith dysfunction was the most found in BPPV. LSCC, lateral semicircular canal; VN, vestibular neuritis; MD, Meniere's disease; RV, recurrent vestibulopathy; VM, vestibular migraine; BPPV, benign paroxysmal positional vertigo.

Lateral canal dysfunction was most common in VN, followed by definite MD, RV, definite VM, and BPPV patients in decreasing order (Figure 3). Otolith dysfunction was the most common in definite MD, followed by VN, RV, BPPV, and definite VM cases in decreasing order. Lateral canal dysfunction was more common than otolith dysfunction in VN patients (*p* < 0.001). However, the other vestibular disorders showed higher otolith dysfunction than lateral canal dysfunction and this difference was significant in patients with BPPV (*p* < 0.01).

### Isolated Otolith Dysfunction in Vestibular Disorders

Isolated otolith dysfunction without lateral canal dysfunction was the most found in BPPV, followed by definite VM, RV, and definite MD in decreasing order (Figure 3). There were no VN patients with isolated otolith dysfunction because the diagnostic definition of VN should include lateral canal dysfunction.

## Discussion

In our present analyses, the most common diagnoses were RV (26.5%), VM (20.5%), BPPV (17.2%), MD (10.6%), VN (10.3%), autonomic dizziness (7.0%), and central lesions (3.3%), which are similar to previous reports (2, 3, 22–24). The most common six disorders thus comprised 92.1% of our study patients. We found different involvement patterns of vestibular dysfunction in the various vestibular disorders in our current study series. First, we observed that abnormal caloric results were more common than abnormal vHIT in all of the vestibular disorders we analyzed, although this was significant only in RV (40 vs. 17%). Second, otolith dysfunction was more common than lateral canal dysfunction in many vestibular disorders except VN. Third, lateral canal dysfunction was significantly more common than otolith dysfunction in VN. The other disorders showed higher abnormal rates in otolith organs than in the lateral canal, although the difference was only significant in patients with BPPV. Furthermore, different patterns of involvement of otolith organs were observed in our current patient population. An abnormal oVEMP test result was more frequent than an abnormal cVEMP finding in VN, but abnormal cVEMP test result was more frequent than an abnormal oVEMP in MD, although those differences were not significant.

Most of the current study patients from our own neurotologic dizziness clinic had peripheral vestibular disorders with only a few cases (3.3%) showing definite central causes that could explain their dizziness. However, central vestibular disorders can be a frequent cause of dizziness in the emergency department where acute vertigo is encountered frequently. In a previous report from an emergency department of a tertiary referral center, 46% of patients with central vertigo showed associated neurological symptoms with vestibular symptoms, which indicated that more than 50% of patients with central vertigo may not complain of other neurologic symptoms (25). A proper physical examination is critical to diagnosing patients with central vertigo because not all of these cases show neurological symptoms. There is a report that a stroke, which should be diagnosed as early as possible, can be identified by proper examinations: a normal horizontal head impulse test result, direction-changing nystagmus in eccentric gaze, or skew deviation (HINTS: Head-Impulse-Nystagmus-Test-of-Skew) was 100% sensitive and 96% specific for diagnosing stroke in patients with acute vestibular syndrome, although an initial MRI diffusion-weighted imaging analysis was falsely negative in 12% (all <48 h after symptom onset) (16). Although central vertigo is rare in an out-patient clinic, an approach that integrates a detailed history taking, proper examination, and appropriate imaging is essential for its diagnosis and treatment.

It has been reported that there is a differential involvement of caloric and vHIT test results in patients with VN, MD, and VM (8, 26–28). We found that that bi-thermal caloric testing (low frequency vestibular afferent receptor testing) was abnormal in all VN patients and vHIT in 83 percent of cases. This may relate to the timing of the tests after the onset of symptoms. In addition, not only low gain but also presence of corrective saccades should count in the definition of a vHIT abnormality (20, 29, 30). A differential involvement of the cVEMP and oVEMP tests has also been reported in VN and MD (31–36). In patients with VN, we found a higher abnormal rate (50%) in the oVEMP test compared to that (30%) in the cVEMP test, suggesting a higher incidence of utricular dysfunction than saccular dysfunction in VN (37), although this was not significant in this study. This trend was reported previously in a study in which 11 patients (55%) had an abnormal oVEMP finding and 5 cases (25%) had an abnormal cVEMP test among a group of 20 VN patients (31). In contrast however, a higher abnormal rate from a cVEMP compared to an oVEMP test has also been reported in MD patients, suggesting a greater deterioration of the saccule than the utricle (32–34). Similarly, in our present analysis we found a higher abnormal rate (69%) in a cVEMP test compared to that (50%) in an oVEMP test in our definite MD patients and the definite VM cases also showed a similar pattern to that of the definite MD patients. Notably, it has also been reported in another study that both MD and VM behaved similarly on a VEMP test battery (38).

Interestingly, isolated otolith dysfunction was found in 44% of BPPV, 32% in definite VM, and 19% in RV and definite MD. It is reasonable that isolated otolith dysfunction is less found in vestibular disorders with high incidence of lateral canal and otolith dysfunction like VN and MD. It is interesting that BPPV showed higher incidence of isolated otolith dysfunction because the incidence of lateral dysfunction is low (13%) compared to other vestibular disorders, suggesting that vestibular dysfunction could have not been identified if only the caloric or vHIT was performed in BPPV patients. This otolith dysfunction may explain the excessive postural complains that these patients often report.

The significance of these different patterns of involvement of vestibular function tests need to be pursued in a larger population of patients in the future as they may be suggestive of a differential impairment of vestibular organs and help to explain differences and/or commonalities in the pathogenesis of various vestibular disorders and possibly provide a guideline about which target organs the vestibular rehabilitation exercises are applied for.

## Conclusion

The most common diagnoses in patients with dizziness in descending order were RV (26.5%), VM (20.5%), BPPV (17.2%), MD (10.6%), VN (10.3%), orthostatic dizziness (7.0%), and central lesions (3.3%). A combination of caloric, vHIT, cervical and ocular VEMP tests indicated different patterns of involvement on the various vestibular endorgans indicating various pathogenesis in vestibular disorders. Otolith dysfunction was found to be more common than lateral canal dysfunction in many vestibular disorders except VN, which has been neglected by not performing the otolithic function tests.

## Data Availability Statement

The raw data supporting the conclusions of this article will be made available by the authors, without undue reservation.

## Ethics Statement

The studies involving human participants were reviewed and approved by the Institutional Review Board of Asan Medical Center. Written informed consent for participation was not required for this study in accordance with the institutional requirements.

## Author Contributions

YK, BK, MY, and HP: design of the work, interpretation, revising the work, and final approval of the version to be published. YK and BK: acquisition. YK, BK, and MY: analysis. All authors contributed to the article and approved the submitted version.

## Conflict of Interest

The authors declare that the research was conducted in the absence of any commercial or financial relationships that could be construed as a potential conflict of interest.

## Publisher's Note

All claims expressed in this article are solely those of the authors and do not necessarily represent those of their affiliated organizations, or those of the publisher, the editors and the reviewers. Any product that may be evaluated in this article, or claim that may be made by its manufacturer, is not guaranteed or endorsed by the publisher.
